# Multiple recurrences and risk of disease progression in patients with primary low-grade (TaG1) non–muscle-invasive bladder cancer and with low and intermediate EORTC-risk score

**DOI:** 10.1371/journal.pone.0211721

**Published:** 2019-02-27

**Authors:** Marie Simon, Pierre-Olivier Bosset, Mathieu Rouanne, Simone Benhamou, Camelia Radulescu, Vincent Molinié, Yann Neuzillet, Xavier Paoletti, Thierry Lebret

**Affiliations:** 1 Service de Biostatistique et d'Epidémiologie & CESP OncoStat, INSERM, Institut Gustave Roussy, Université Paris Saclay, UVSQ, Villejuif, France; 2 Department of Urology, Hôpital Foch, Université Paris-Saclay, UVSQ, Suresnes, France; 3 INSERM, UMR 946, Genetic Variation and Human Diseases Unit, Paris, France; 4 Department of Pathology, Hôpital Foch, Université Paris-Saclay, UVSQ, Suresnes, France; 5 Department of Pathology, University Hospital of Martinique, Fort-de-France, France; Centro Nacional de Investigaciones Oncologicas, SPAIN

## Abstract

**Aim:**

To assess the prognostic value of multiple recurrences on the risk of progression in a large cohort of TaG1 bladder cancer of low and intermediate risk based on the EORTC score and to evaluate prognostic factors of multiple recurrences.

**Materials and methods:**

We retrospectively analyzed a French cohort of 470 patients with primary TaG1 bladder cancer diagnosed between 1986 and 2010 and followed until 2012. They were classified at low and intermediate risk using the EORTC risk score. Associations between the number of recurrences and the risk of progression to high grade Ta/T1 bladder cancer and progression to muscle-invasive disease were assessed. The characteristics of recurrences, as occurrence time or localization, and risk of other recurrences were evaluated.

**Results:**

Out of 470 patients, 251 had recurrence, 34 progressed to high grade Ta/T1 and 17 to muscle-invasive disease, including 4 who had non muscle-invasive progression first. The median follow-up was 7.2 years (interquartile range: 4.2–10.9). In half the progressions, no previous recurrence was observed. No association between the number of recurrences and the risk of progression was detected. Even after 5 years free of event, patients had a 15% risk of recurrence. History of two or more recurrences increased by 4.5 the risk of subsequent recurrence. Time between two recurrences inferior to six months and multifocal localization increased the risk of recurrence.

**Conclusion:**

Surveillance of patients with TaG1 should be continued beyond 5 years of follow-up. However, cystoscopy exams could be spaced after 5 years. Multiple TaG1 recurrences did not appear to be prognostic for disease progression, but increased significantly the risk of subsequent recurrences. Short time between two recurrences and multifocal localization may serve to adapt monitoring of patients with TaG1 Bladder cancer.

## Introduction

Bladder cancer (BC) is the 9^th^ most commonly diagnosed cancer in the world [1]. The worldwide age standardized incidence rate (per 100,000 person-years) was 9.0 for men and 2.2 for women in 2012 [1]. In France, the incidence is increasing by about 1% per year [2–3]. Approximately 75–85% of BC are non-muscle-invasive. Of these, 70% are stage Ta, 20% are T1 and 10% are in situ (Cis) [4–7]. Ta papillary tumors with histopathological grade (G) 1 (or low grade) are those most frequently found at diagnosis. The natural history of superficial bladder cancer is characterized by a high rate of recurrence (between 30% and 78% at 5 years) in the same stage/grade but a limited five-year risk of progression to muscle invasive disease of stages T2 to T4 (7% to 40%) [8–11]. These recurrent events alter the patient’s quality of life and might increase the risks of progression either to a High-grade Ta, T1, CIS, or to T2-T4 pathological stage, which in turns have a major impact on the therapeutic management.

American and European studies have identified baseline prognostic factors of recurrence and/or progression [7–13]. Tumor size (> 30 mm), pathological stage T1, high grade, presence of Cis, number of lesions (multiple lesions), history of recurrence during the first year after diagnosis, and advanced age appeared to increase the risk of recurrence and/or progression. The European Organization for Research and Treatment of Cancer (EORTC) developed in 2006 a scoring system for Ta-T1 BC to predict the risk of progressions to muscle-invasive disease based on data from 7 clinical trials [8]. The European Association of Urology (EAU) proposed to categorize patients with non muscle invasive BC into three risk groups identified from this score [4], to facilitate treatment recommendations. Based on the 2018 EAU guidelines, regular monitoring via cystoscopy is recommended in low-risk TaG1 patients. Immediate post-operative intravesical instillation of chemotherapy (IPOIC) may be added at all risk levels. For the other risk groups, a medical treatment of several months should be discussed: intravesical Mitomycin C or BCG instillations for intermediate-risk patients, and BCG instillations for high-risk patients [4].

The associations between recurrence and progressions to higher stage / grade or to muscle-invasive disease are poorly documented in the low grade (TaG1) population. Furthermore, recurrence rate is most often measured at baseline and is not updated with the occurrence of new recurrences [7–10, 12–13]; the link between tumor characteristics and the risk of subsequent recurrences, taking into account the whole patient history of recurrences after the first transurethral resection of a bladder tumor (TURB), has been poorly investigated so far. Finally, tumor evolution in homogeneous cohorts of patients with primary TaG1 and long-term follow-up are lacking.

The objectives of this study were to investigate the association between the number of previous recurrences and progression to high grade Ta/T1 and to muscle invasive disease in the TaG1 population at low or intermediate risk, to validate prognostic factors of recurrence and to investigate the inter-relationship between repeated recurrences, time to recurrence, or multifocal localization, after TURB diagnosis.

New insights on the natural history of TaG1 papillary tumors may improve our ability to adapt the follow-up of those patients who are considered at low-risk by the EAU classification.

## Methods

### Study population

This is a French retrospective observational cohort of primary consecutive TaG1 pure urothelial BC diagnosed and treated between 1986 and 2010 in Foch hospital [9]. The Gustave Roussy Cancer Center ethic committee (CSET) approved this research. No signed informed consent was requested as the study was purely observational. Individual data of patients, who underwent a first TURB and a macroscopic radical resection (possibly after two interventions), were collected from medical and anesthesiologist records. A cut-off date was set at 15/01/2012.

Tumor stage and grade were determined by two uropathologists with a central review according to the TNM and grade classification (1973 World Health Organization classification) to include solely initial diagnosis of TaG1 BC. Patients with an upper urinary tract tumor or primary concomitant Cis were excluded [14, 15]. The tumor grade of events that occurred during follow-up was reviewed by two uropathologists (VM, CR) and reclassified using the 2004 WHO classification: G1 and G3 were classified in low and high grade respectively. All G2 cases were classified in low or high-grade, taking into account clinical context when necessary. Cis status was systematically reclassified in high-grade disease. Baseline was taken as the date of TURB. Patients were followed according to the French Association of Urology guidelines: Regular monitoring included cytology and flexible cystoscopy performed three months after initial TURB, then every trimester for two years, every semester thereafter until five years, and then yearly. Computed tomographic scan examination were done every other year for upper-urinary tract check-up [9]. All patients who had a suspicion of disease recurrence underwent an additional TURB: detrusor in the specimen were observed for all T1 or T2 recurrences as standard practice included some muscle resection but no fulguration was performed; recurrences were confirmed by a uropathologist and treatments were collected. No patient received any adjuvant treatment after the initial TaG1 diagnosis prior to the first recurrence (no IPOIC, BCG, or mitomycin C instillations). For patients who discontinued the follow-up at Foch hospital, the new treating urologists were contacted to update patients’ clinical status; however recurrences or progression were then not centrally reviewed. The management of tumor recurrence followed the EAU recommendations [4].

### Endpoints

Time to progression to higher grade Ta / T1 was defined as the time from diagnosis to the date of first increase to T1 stage or high grade/G3 tumors or Cis. Time to progression to muscle-invasive disease was defined as the time from diagnosis to the date of first increase to T2 stage or higher. Patients still alive and without disease progression at the cut-off date were censored. The primary endpoint was "all types of progression" i.e. high-grade Ta or T1 to T4 progression whichever came first. The time to first recurrence was defined as a new TaG1 or Ta low grade/G2 tumor in the same way. Deaths without tumor progression were censored at the date of death and not considered as an event. When investigating the risk of the first recurrence, we ignored progression before recurrence, as it rarely occurred.

### Prognostic factors of progression and/or recurrence

Data on size and number of tumors was collected from TURB reports. Tumor site (including multifocal status) and the type of care (surveillance, type of adjuvant treatment or surgery) were available at baseline and during follow-up though standardized case report forms filled in by surgeons. Smoking status (smoker or non-smoker) was collected from anesthesiologist medical records at baseline [16].

### Longitudinal statistical analysis

The median follow-up was estimated using the inverse Kaplan Meier method [17]. Log-rank test and Cox model analysis were performed to identify prognostic factors of recurrence and progressions. The cut-off values for the categorical variables came from the univariate analyses or literature data. No model selection was performed and all multivariate models were adjusted on: age, sex, smoking status, treatment received during follow-up (yes or no), and tumor characteristics such as number of tumors (1, 2–7, >7), size (≤30 mm or >30 mm). To analyze the risk of progressions to higher grade Ta or to muscle invasive diseases, we accounted for multiple recurrences occurring in the same patient (explanatory variable) using multivariate time dependent Cox model. To analyze the effect of the number of recurrences on the risk of subsequent recurrence, landmark was used in order to correct for the “selection bias” [18,19]: In this analysis, baseline time was postponed to 3 years at which 50% of the recurrences had occurred, and at later timepoints. Finally, to investigate the impact of the localization of recurrence number k and the risk of recurrence k+1 as well as the delay between two recurrences, we used multivariate frailty models, which produce hazard ratios and confidence intervals as association measures [20,21]. As a sensitivity analysis, we excluded recurrences that occurred less than 3 months after TURB, which could be the result of an incomplete resection of the primary tumor. All statistical analyses were performed using SAS 9.4.

## Results

### Study population

**Fig 1:**
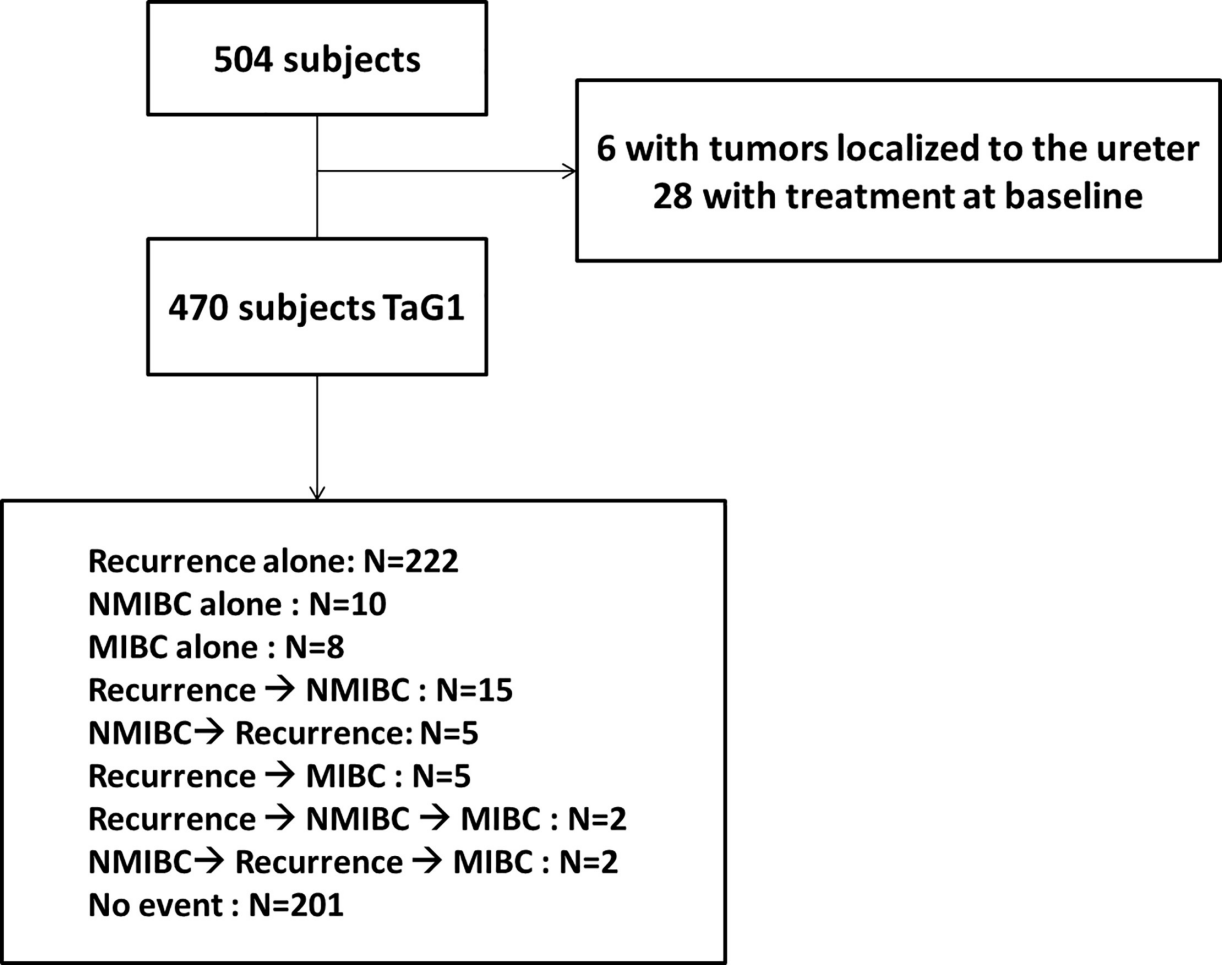
Flow chart.

Between April 1986 and December 2010, 504 consecutive patients underwent a TURB for newly diagnosed TaG1 BC. Thirty-four patients were subsequently excluded because of upper-tract tumor localization (n = 6) and treatment at baseline (n = 28) leading to a study population of 470 TaG1 (Fig 1). Mean age of patients was 66 years. Sex-ratio (M/F) was 4.3:1. Overall, 70% of the population were smokers and 59% were at low-risk of progression to muscle-invasive disease according to the EORTC score (Table 1). The median follow-up was 7.20 years (interquartile range: 4.20–10.88 years). Follow-up was discontinued after 10 years by standard practice and 65 were lost-to follow-up, i.e. no data could be collected for their last planned visit. Recurrence occurred in 251 patients. In total, the median number of recurrences per patient was 1 (range: 0–19). Progression to T1 or TaG3 cancer occurred in 34 patients and progression to muscle-invasive disease occurred in 17 patients, including 4 who had non muscle-invasive progression first. Respectively 17 and 8 of them occurred despite the absence of previous TaG1 recurrence events (Fig 1). The characteristics of the 47 patients who progressed are listed in Text A in S1 File. During the follow-up, 201 patients had no relapse or progression events and 73 patients died (including 59 patients who never experienced disease progression).

**Table 1:** Baseline characteristics of patients and tumors TaG1.

**Mean (sd)** **N (%)**		**STUDY POPULATION** **N = 470**	**MISSING**
**Age (years)**		65.74 (12.5)	-
**Age in class**	< = 60	154 (32.8)	-
	61–70	135 (28.7)	
	71–80	146 (31.1)	
	>80	35 (7.5)	
**Sex**	Men	381 (81.1)	-
	Women	89 (18.9)	
**Smoking**	Yes	333 (72.1)	8 (1.7)
**EORTC score**	Low risk	277 (58.9)	-
	Intermediate risk	193 (41.1)	
**Size**	<30mm	385 (81.9)	1 (0.2)
	>30 mm	84 (17.9)	
**Number**	1	338 (71.9)	1 (0.2)
	2–7	101 (21.5)	
	>7	30 (6.4)	
**Localization**	DOME	34 (7.2)	3 (0.6)
	FLD	108 (23.0)	
	FLG	115 (24.5)	
	FLD & FLG	2 (0.4)	
	TRIG	43 (9.2)	
	OUD/OUG	120 (25.5)	
	COL	14 (3.0)	
	Multifocal	31 (6.6)	
**Treatment**	Surveillance	463 (98.5)	-
	2^nd^ look	7 (1.5)	

### Prognostic factors of progression

**Fig 2:**
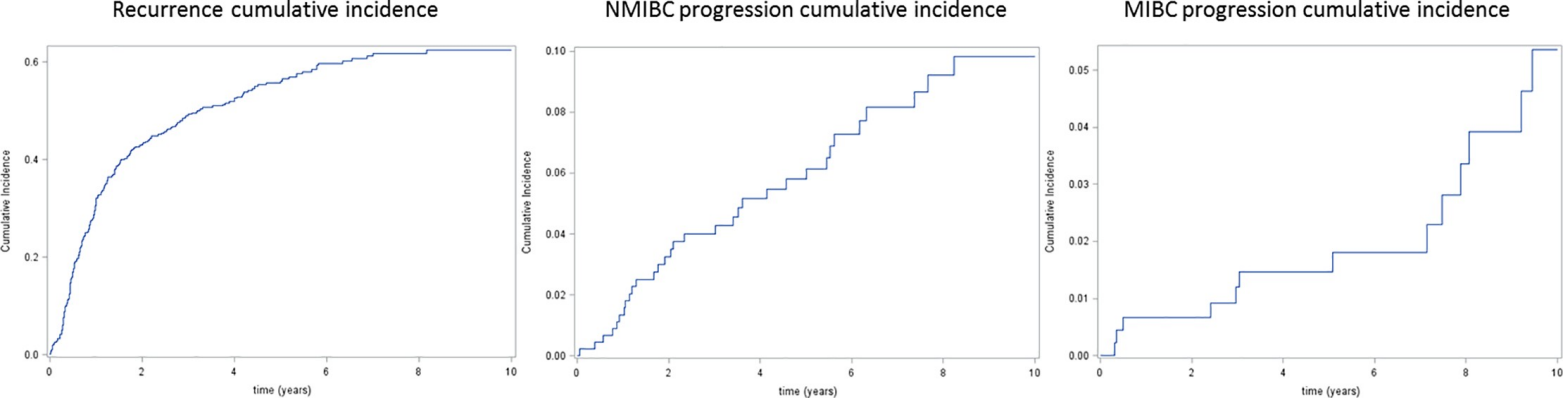
Cumulative incidence curves. Bladder cancer Recurrence time, progression to high grade Ta / T1 (NMIBC) time; Muscle invasive (MIBC) progression time.

At seven years, the cumulative incidence of progression to high grade TaT1 disease and to muscle-invasive disease were 8.2% (95%CI: 4.8–10.5) and 1.8% (95%CI: 0–2.7) respectively (Fig 2 and Figure A Panels A.1, A.2 and A.3 in S1 File). Whatever the type of first progression considered no statistically significant baseline prognostic factor was found. Prognostic value of recurrences that occurred over time is summarized in Table 2 (Tables A and B in S1 File). The number of previous recurrences was not a significant prognostic factor for the outcome “all types of progression” even after multiple recurrences. Similar results were obtained when considering only progression to high grade Ta/T1 disease. Results were similar after the exclusion of the 20 patients who recurred within 3 months after the initial TURB (See Table C in S1 File).

**Table 2:** Results of univariate and multivariate analyzes of the effect of the number of recurrences on progression-free survival—variable time-dependent cox model.

** **		BOTH PROGRESSION FREE TIME *		PROGRESSION TO HIGH GRADE Ta / T1 FREE TIME	
		HR (CI 95%)		HR (CI 95%)	
		Univariate	Multivariate ƚ	Univariate	Multivariate ƚ
**Number of recurrences**	1	1.50 (0.72–3.11)	0.92 (0.41–2.06)	2.04 (0.88–4.75)	0.90 (0.33–2.46)
	2–3	1.47 (0.64–3.39)	0.90 (0.36–2.23)	2.66 (1.07–6.61)	1.51 (0.54–4.10)
	> = 4	0.95 (0.31–2.90)	0.51 (0.15–1.69)	0.91 (0.18–4.52)	0.43 (0.07–2.53)

* Both type of progression: NMIBC or MIBC.

Univariate and multivariate hazard ratios were estimated using variable time-dependant cox model.

ƚ Adjusted on age, sex, smoking, previous treatment and stratified on the EORTC risk score (low versus moderate/high) at baseline.

### Prognostic factors of recurrence

**Fig 3:**
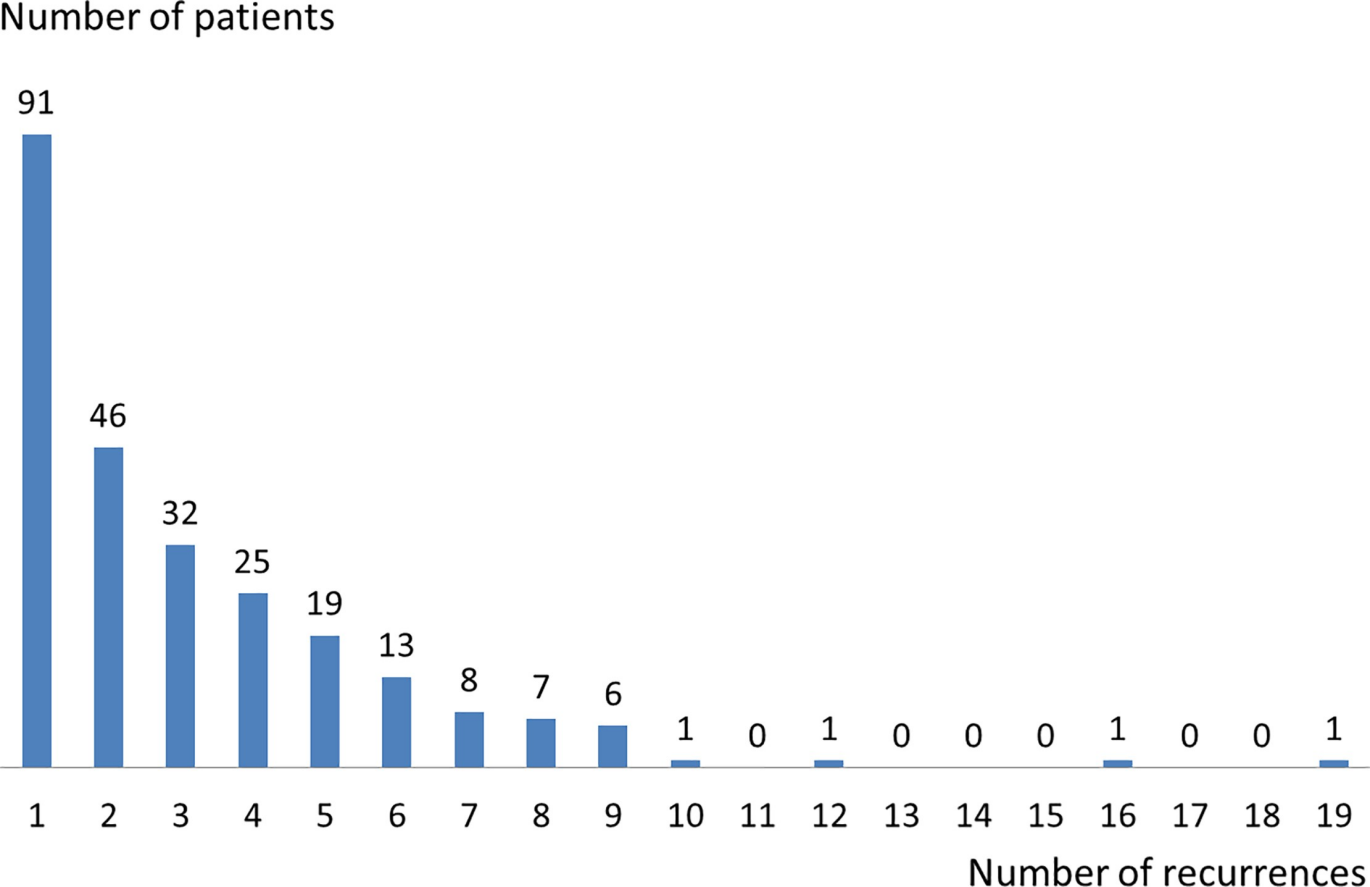
Distribution of patients according to the number of recurrences occurring during follow-up.

Fig 3 illustrates the number of recurrences per patient (median = 1, range 0–19). Tumors were more often multifocal at baseline than at recurrences (17.9% vs. 6.1%). Therapeutic management of recurrences was IPOP (14%), mitomycin (7%) and BCG (12%). Surveillance was maintained after 63% of the recurrences. As shown on Fig 3, the median recurrence-free time was 3.2 years (95%CI: 2.3–4.4) and incidence peaked before three years (Figure A Panel A.1 in S1 File). The cumulative incidence of first recurrence reached 60% at seven years. When we focus on patients who were free of recurrence at 2 years, the cumulative risk of recurrence over the 4 following years was 30% and patients with no recurrence at 5 years had still a 15% risk of developing a first recurrence between years 5 and 9 (Table B in S1 File).

Baseline prognostic factors associated with the risk of first recurrence were: the size of the tumor (p<0.001) and the number of lesions (p <0.001). After adjustment, patients relapsing once in the three first year after TURB were more likely to have subsequent recurrence compared to those who did not (HR = 2.11; 95%CI: 1.28–3.49) as were patients with 2 or more recurrences (HR = 4.72; 95%CI: 2.77–8.05) (Table 3 and Table C in S1 File). History of recurrences in the last 6 months increased by 33% the risk of subsequent recurrence (Table 4 and Table D in S1 File). Low intra-patient correlation was found between repeated recurrences, which suggested that the times before recurrence were relatively independent of each other. Finally, the multifocal localization (HR = 1.47; 95%CI: 1.19–1.80) was significantly associated with the recurrence-free time (Table 3 and Table D in S1 File).

**Table 3:** Results of univariate and multivariate analyzes of the effect of number of recurrences on recurrence-free time–Landmark analysis.

			RECURRENCE FREE TIME	
Landmark	Number of		HR (CI 95%)	
** **	recurrences	Effectives	Univariate	Multivariate ƚ
**3 YEARS**	1	90	2.21 (1.39–3.51)	2.11 (1.28–3.49)
	2–3	80	4.94 (3.25–7.50)	4.72 (2.77–8.05)
	> = 4	15	3.13 (1.39–7.04)	2.63 (0.97–7.15)

Univariate and multivariate hazard ratios were estimated using cox model.

ƚ Adjusted on age, sex, smoking, previous treatment and stratified on the EORTC risk score (low versus moderate/high) at baseline.

**Table 4:** Results of univariate and multivariate analyzes of the effect of recurrences characteristics on recurrence-free time–Frailty model.

** **		RECURRENCE FREE TIME	
		HR (CI 95%)	
		Univariate	Multivariate ƚ
**Localization**	Multifocal	1.53 (1.26–1.86)	1.47 (1.19–1.80)
**Time of occurrence**	< 6 months	1.38 (1.12–1.70)	1.33 (1.08–1.63)

Univariate and multivariate hazard ratios estimated from Frailty models; time of recurrence was estimated on the patients having at least one recurrence.

ƚ Adjusted on age, sex, smoking, previous treatment and on the EORTC risk score (low versus moderate/high) at baseline.

## Discussion

In this large single-center cohort of 470 primary TaG1 low and intermediate risk BC with long-term follow-up, we found that half of the patients with disease progression did not experience any prior tumor recurrence resulting in the absence of association between the two outcomes, even for patients with multiple (>4) recurrences. However, history of 2 or more recurrences increased by 4.5 the risk of subsequent recurrence. Even 5 years free of recurrence after the initial TURB, patients had a 15% risk of developing a new recurrence, suggesting that follow-up should be maintained on a long-term period. Indeed, we also found that previous recurrence within the last 6 months was prognostic of new recurrence. Finally, we confirmed that multifocal localization at diagnosis was a risk factor of subsequent recurrence [7–13].

Overall, the frequent cystoscopic examinations entail a heavy cost to the society, and BC induces the highest cost per patient from diagnosis to death among cancer types [22]. The possibility to reassure TaG1 patients with multiple recurrences regarding the risk of progression and to increase the interval between two cystoscopy exams in low risk patients both motivate the identification of prognostic factors. American and European studies that focused on baseline prognostic factors of recurrence and/or progression [7–13] reported that prior recurrence rate (<1/year, > 1/year) or the history of recurrence (>1 vs 0, without precision of time of occurrence) were prognostic of recurrence and of progression. However, inclusion criteria allowed a prior history of BC; the presence of T1 pathological substage, or adjuvant treatment after the initial TURB. All these parameters increase the heterogeneity in study population and strongly impact the clinical outcome of the patients. Few studies have investigated the impact of repeated recurrences on the risk of progression. Lujan et al. accounted for multiple recurrences [11] and concluded to a mild association between these two risks but this study presented the same heterogeneity regarding the inclusion criteria as discussed previously. Recently, Golabesk (2017) reported that among a single-institution retrospective cohort of 704 patients including 548 Ta patients (median follow-up = 5 years), only 3.6% of the initial tumors relapsed after 5 years of follow-up, which was lower than the predicted values from the EORTC score [23]. In Balan (2018), long-term survival of a homogeneous cohort of 164 Ta low grade patients was similar between G1 and G2 tumors but they did not investigate timing of events [24].

Although EORTC risk tables provide a prognostic tool, no molecular biomarkers accurately predict disease progression. In recent years, the hypothesis of “distinct biological pathway” has been proposed to explain the different evolution between NMIBC [5,25–27]. This hypothesis involved mutation of tumor suppressor genes, like P53, or oncogene deletion like FGFR3 that would characterize two distinct genetic pathways associated either to multiple recurrences or to progression. However, mutation patterns have not yet been identified to consistently discriminate between patients with low or high-risk of disease progression. In addition, no molecular biomarker has been implemented in the clinical practice. Recently, Hedegaard et al performed a comprehensive transcriptional analysis in a large cohort of 460 primary Ta/T1 bladder cancers and found that they could be subgrouped into 3 major molecular classes with basal and luminal-like characteristics, with widely different outcomes [28]. Large prospective cohort studies, collecting repeated epidemiological, clinical, pathological, and molecular data, such as the COBLANCE study will be key to explore the relationships between epidemiological, clinical, pathological, and molecular data.

The major strengths of our study include a long median follow up period of more than seven years. Number of events was substantial and provided an important power for our statistical analysis of risk of recurrence. Moreover, the study population was homogeneous in terms of treatment management, which reduces potential confounding factors. Our study is one of the first that took into account the entire clinical history of the patients after TURB.

The principal limitations of this cohort are related to its retrospective nature and the risk of selection bias. The follow-up and the clinical management corresponded to daily clinical practice. Therefore, modifications of the evaluation of patients may have occurred. This heterogeneity is somehow reduced by the fact that the same team staged and treated all the patients, which improved the standardization of the treatment of non-muscle invasive recurrences. Despite we adjusted analyses on the received treatment for recurrence, variations over years might have affected the risk of progression. Furthermore, despite a cut-off date at which status of all patients was queried via their treating urologist, there was still 14% of lost-to-follow up patients. For the patients who were followed-up outside of the Foch hospital, no imaging data was collected to confirm recurrences or progression reported by the treating physician. A second important limitation is the insufficient statistical power to investigate the progression to muscle-invasive disease and the modification of this risk over time. Finally we lack epidemiological data. For instance, smoking status was reported as a binary (yes/no) variable but no data was available regarding quantity and duration of smoking which may increase the risk of recurrence and progression [16]. Another potentially important missing variable was the Ki 67, a marker of cell proliferation that has been associated with shorter time to recurrence and time to progression [29]. A meta-analysis of TaG1 cohorts should be carried out to have enough power to investigate the prognostic value of recurrences on the risk of progression to muscle invasive diseases.

## Conclusion

The number of low-grade (TaG1) recurrences did not appear to be prognostic for disease progression, but increased significantly the risk of subsequent recurrences as a short time to recurrence and multifocal localization did. Therefore, surveillance of patients with TaG1 tumors at low / intermediate risk based on the EORTC score might be adapted based on those simple variables. It should be continued beyond 5 years of follow-up as 17 of the 47 progressions occurred 5 years after initial TURB and the hazard of any progressions remained relatively high but yearly cystoscopy could be recommended instead of 6-monthly exams to account for the decreasing risk of recurrence.

## Supporting information

S1 File Supporting information. **Figure A**: Hazard functions for recurrence (A.1), progression to Ta/T1 stage (A.2) or progression to muscle invasive bladder cancer (A.3). Fig A represents the hazard function for the first recurrence, which is the rate at which new events occur in the at-risk population; it was maximal before one year after diagnosis and then decreased progressively over time; at three years, the cumulative incidence of recurrence was close to 0.7. Fig B represents the hazard function for the progression to Ta/T1 stage bladder cancer or non-muscle invasive bladder cancer (NMIBC); it decreased continuously over time. Fig C represents the hazard function for the progression to muscle invasive bladder cancer (MIBC). The hazard risk of muscle invasive bladder cancer progression was low in the first year after diagnosis and then increased thereafter.
**Table A:** Sensitivity analysis of the risk of progression after exclusion of the 20 patients who relapsed within the 3 months after the initial TURB. Table A: Multivariate Hazard Ratios (95% Confidence Intervals) of the effect of the number of recurrences on both progression-free time with details on covariates–Variable time-dependant cox model. Table B: Multivariate Hazard Ratios (95% Confidence Intervals) of the effect of the number of recurrences on NMIBC progression-free time with details on covariates–Number of recurrence is a time-dependant variable in the cox model. Table C: Univariate and multivariate analyzes of the effect of the number of recurrences on progression-free survival.
**Table B:** Cumulative incidence of first recurrence over the 4 years in patients free of recurrence at increasing timepoints.
**Table C**: Effect of the number of previous recurrences on the risk of subsequent recurrence–Landmark analysis at 3 year.
**Table D:** Multivariate Hazard Ratios (95% Confidence Intervals) of the effect of recurrences’ characteristics on recurrence-free time with details on covariates–Frailty models.
**Text A** List of the characteristics of the 47 patients who progressed to higher grade or stage during the follow-up.
(DOCX)

## References

[1] Ferlay J, Soerjomataram I, Dikshit R, Eser S, Mathers C, Rebelo M, Parkin DM, Forman D, Bray F. Cancer incidence and mortality worldwide: sources, methods and major patterns in GLOBOCAN 2012. Int J Cancer. 2015 3 1;136(5):E359–86. 10.1002/ijc.29210 25220842

[2] Rouprêt M, Neuzillet Y, Masson-Lecomte A, Colin P, Compérat E, Dubosq F, Houédé N, Larré S, Pignot G, Puech P, Roumiguié M, Xylinas E, Méjean A. CCAFU french national guidelines 2016–2018 on bladder cancer. 10.1016/S1166-7087(16)30703-5 27846934

[3] Pfister C1, Roupret M, Wallerand H, Davin JL, Quintens H, Guy L, Houede N, Bernardini S, Larré S, Mazerolles C, Roy C, Amsellem D, Saint F, Irani J, Soulié M. Recommendations Onco-Urology 2010: Urothelial tumors. Prog Urol. 2010 11; 20 Suppl 4:S255–74. 10.1016/S1166-7087(10)70043-9 21129645

[4] Babjuk M, Burger M, Compérat EM, Gontero P, Mostafid J, Palou J, van Rhijn BW, Rouprêt M, Shariat SF, Sylvester RJ, Zigeuner R. *EAU Guidelines on Non-muscle-invasive Bladder Cancer (TaT1 and CIS) 2018*, in *European Association of Urology Guidelines* 2018 Edition 2018, European Association of Urology Guidelines Office: Arnhem, The Netherlands Available from: http://uroweb.org/guidelines/non-muscle-invasive-bladder-cancer/

[5] Kamat MA, Hahn NM, Efstathiou JA, Lerner SP, Malmström PU, Choi W, Guo CC, Lotan Y, Kassouf W. Bladder cancer. Lancet. 6 23, 2016 10.1016/S0140-6736(16)30512-8 27345655

[6] Van Rhijn BW, Burger M, Lotan Y, Solsona E, Stief CG, Sylvester RJ, Witjes JA, Zlotta AR. Recurrence and progression of disease in non-muscle-invasive bladder cancer: from epidemiology to treatment strategy. Eur Urol. 2009 9;56(3):430–42. 10.1016/j.eururo.2009.06.028 Review. 19576682

[7] Volmer RT. A Review of Outcomes for Stage Ta Bladder Tumors. Am J Clin Pathol. 2016 8;146(2):215–20. 10.1093/ajcp/aqw103 27473739

[8] Sylvester RJ, van der Meijden APM, Oosterlinck W, Witjes JA, Bouffioux C, Denis L, Newling DWW, Kurth K. Predicting recurrence and progression in individual patients with stage Ta T1 bladder cancer using EORTC risk tables: a combined analysis of 2596 patients from seven EORTC trials. Eur Urol 2006;49: 466–77. 10.1016/j.eururo.2005.12.031 16442208

[9] Bosset O, Neuzillet Y, Paoletti X, Molinie V, Botto H, Lebret T. Long-term follow-up of TaG1 non-muscle-invasive bladder cancer. Urol Oncol. 2015 1;33(1):20.e1–7. 10.1016/j.urolonc.2014.09.001 25282704

[10] Rieken M, Xylinas E, Kluth L, Crivelli J, Chrystal J, Faison T, Lotan Y, Karakiewicz PI, Holmang S, Babjuk M, Fajkovic H, Seitz C, Klatte T, Pycha A, Bachlabb A, Scherr DS, Shariat SF. Long-term cancer-specific outcomes of TaG1 urothelial carcinoma of the bladder. EurUrol 2014;65:201– 10.1016/j.eururo.2013.08.034 23998688

[11] Luján S, Santamaria C, Pontones JL, Ruiz-Cerdá JL, Trassierra M, Vera-Donoso CD, Solsona E, Jiménez-Cruz F. Risk estimation of multiple recurrence and progression of non muscle invasive bladder carcinoma using new mathematical models. Actas Urol Esp. 2014. 10.1016/j.acuro.2014.04.007 24930059

[12] Zieger K, Wolf H, Olsen PR, Hojgaard K. Long-term follow-up of noninvasive bladder tumours (stage Ta): recurrence and progression. BJU International (2000), 85, 824±828. 10792160 10.1046/j.1464-410x.2000.00547.x

[13] Fernandez-Gomez J, Solsona E, Unda M, et al Prognostic factors in patients with non–muscle-invasive bladder cancer treated with bacillus Calmette-Guerin: multivariate analysis of data from four randomized CUETO trials. Eur Urol 2008;53:992–1002. 10.1016/j.eururo.2007.10.006 17950987

[14] Guy L, Savareux L, Molinié V, Botto H, Boiteux JP, Lebret T. Should bladder biopsies be performed routinely after bacillus Calmette-Guérin treatment for high-risk superficial transitional cell cancer of the bladder? Eur Urol. 2006 9;50(3):516–20 10.1016/j.eururo.2006.03.022 16632184

[15] Le Goux C, Pignot G, Amsellem-Ouazana D, Vieillefond A, Peyromaure M, Flam T, Debré B, Zerbiba M. Pronostic value of ureteral location of upper tract urinary carcinoma. Progrès en urologie 2013 23, 399–404 10.1016/j.purol.2013.01.025 23628098

[16] Aveyard P, Adab P, Cheng K.K, Wallace D.M, Hey K, Murphy M.F: Does smoking status influence the prognosis of bladder cancer? A systematic review. BJU Int., 2002; 90: 228–239. 12133057 10.1046/j.1464-410x.2002.02880.x

[17] Schemper M, Smith TL. A note on quantifying follow-up in studies of failure time. Control Clin Trials. 1996 8;17(4):343–6. 8889347 10.1016/0197-2456(96)00075-x

[18] Anita Giobbie-Hurder, Gelber Richard D., and Regan Meredith M. Challenges of Guarantee-Time Bias. Clin Oncol 31:2963–2969 10.1200/JCO.2013.49.5283 PMC3732313 23835712

[19] Dafni Urania. Landmark Analysis at the 25-Year Landmark Point. Circ Cardiovasc Qual Outcomes. 2011;4:363–371 10.1161/CIRCOUTCOMES.110.957951 21586725

[20] Vaupel JW, Manton KG, Stallard E. The impact of heterogeneity in individual frailty on the dynamics of mortality. Demography. 1979 8;16(3):439–54. 510638

[21] Gui-shuang Y, Chengcheng L. Statistical Analysis of Clustered Data using SAS® System.

[22] Hong YM, Loughlin KR. Economic impact of tumor markers in bladder cancer surveillance. Urology. 2008 1;71(1):131–5. 10.1016/j.urology.2007.08.014 18242381

[23] Golabesk T, Palou J, Rodriguez O, Parada R, Skrobot S, Peña JA, Villavicencio H. Long-term Bladder and Upper Urinary Tract Follow-up Recurrence and Progression Rates of G1-2 Non-muscle-invasive Urothelial Carcinoma of the Bladder. Urology. 2017 2;100:145–150. 10.1016/j.urology.2016.07.063 Epub 2016 Oct 17. 27765584

[24] Balan D, Martha O, Chibelean CB, Tataru S, Voidezan S, Sin A, Matei VD, Vartolomei MD, Lucarelli G, Cioffi A, Del Giudice F, De Berardinis E, Borda A, Busetto GM, Ferro M, Pytel A, Porav-Hodade D. Comparison of 10-year overall survival between patients with G1 and G2 grade Ta bladder tumors. Medicine (Baltimore). 2018 4;97(16):e0522 10.1097/MD 29668641 PMC5916673

[25] Knowles MA. Role of FGFR3 in urothelial cell carcinoma: biomarker and potential therapeutic target. World J Urol 2007;25:581–93. 10.1007/s00345-007-0213-4 17912529 PMC4876910

[26] Hernandez S, Lopez-Knowles E, Lloreta J, Kogevinas M, Amoros A, Tardon A, et al Prospective study of FGFR3 mutations as a prognostic factor in nonmuscle invasive urothelial bladder carcinomas. J Clin Oncol 2006;24:3664–71. 10.1200/JCO.2005.05.1771 16877735

[27] Van Rhijn BW, Zuiverloon TC, Vis AN, Radvanyi F, van Leenders GJ, Ooms BC et al Molecular grade (FGFR3/MIB-1) and EORTC risk scores are predictive in primary non-muscle-invasive bladder cancer. Eur Urol 2010;58:433–41. 10.1016/j.eururo.2010.05.043 20646825

[28] Hedegaard J, Lamy P, Nordentoft I, Algaba F, Høyer S, Ulhøi BP, Vang S, Reinert T, Hermann GG, Mogensen K, Thomsen MBH, Nielsen MM, Marquez M, Segersten U, Aine M, Höglund M, Birkenkamp-Demtröder K, Fristrup N, Borre M, Hartmann A, Stöhr R, Wach S, Keck B, Seitz AK, Nawroth R, Maurer T, Tulic C, Simic T, Junker K, Horstmann M, Harving N, Petersen AC, Calle ML, Steyerberg EW, Beukers W, van Kessel KEM, Jensen JB, Pedersen JS, Malmström PU, Malats N5, Real FX, Zwarthoff EC, Ørntoft TF, Dyrskjøt L. Comprehensive Transcriptional Analysis of Early-Stage Urothelial Carcinoma. Cancer Cell. 2016 7 11;30(1):27–42. 10.1016/j.ccell.2016.05.004 Epub 2016 Jun 16. 27321955

[29] Tian Y, Ma Z, Chen Z, Li M, Wu Z, Hong M, Wang H, Svatek R, Rodriguez R, Wang Z. (2016) Clinicopathological and Prognostic Value of Ki-67 Expression in Bladder Cancer: A Systematic Review and Meta-Analysis. PLoS ONE 11(7): e0158891 10.1371/journal.pone.0158891 27410033 PMC4943634

