# Supplementary material for: Multiple recurrences and risk of disease progression in patients with primary low-grade (TaG1) non–muscle-invasive bladder cancer and with low and intermediate EORTC-risk score
Source: PLoS One. 2019 Feb 27;14(2):e0211721. doi: 10.1371/journal.pone.0211721 (PMC6392242; doi:10.1371/journal.pone.0211721)
Supplement: S1 File — Figure A: Hazard functions for recurrence (A.1), progression to Ta/T1 stage (A.2) or progression to muscle invasive bladder cancer (A.3). Fig A represents the hazard function for the first recurrence, which is the rate at which new events occur in the at-risk population; it was maximal before one year after diagnosis and then decreased progressively over time; at three years, the cumulative incidence of recurrence was close to 0.7. Fig B represents the hazard function for the progression to Ta/T1 stage bladder cancer or non-muscle invasive bladder cancer (NMIBC); it decreased continuously over time. Fig C represents the hazard function for the progression to muscle invasive bladder cancer (MIBC). The hazard risk of muscle invasive bladder cancer progression was low in the first year after diagnosis and then increased thereafter. Table A: Sensitivity analysis of the risk of progression after exclusion of the 20 patients who relapsed within the 3 months after the initial TURB. Table A: Multivariate Hazard Ratios (95% Confidence Intervals) of the effect of the number of recurrences on both progression-free time with details on covariates–Variable time-dependant cox model. Table B: Multivariate Hazard Ratios (95% Confidence Intervals) of the effect of the number of recurrences on NMIBC progression-free time with details on covariates–Number of recurrence is a time-dependant variable in the cox model. Table C: Univariate and multivariate analyzes of the effect of the number of recurrences on progression-free survival. Table B: Cumulative incidence of first recurrence over the 4 years in patients free of recurrence at increasing timepoints. Table C: Effect of the number of previous recurrences on the risk of subsequent recurrence–Landmark analysis at 3 year. Table D: Multivariate Hazard Ratios (95% Confidence Intervals) of the effect of recurrences’ characteristics on recurrence-free time with details on covariates–Frailty models. Text A List of the characteristics of the [file pone.0211721.s001.docx]

**Multiple recurrences and risk of disease progression in patients with primary TaG1 non–muscle-invasive bladder cancer**

Marie Simon ^1^ , Pierre-Olivier. Bosset ^2^, Mathieu Rouanne ^2^, Simone Benhamou ^3^ , Camelia Radulescu ^4^, Vincent Molinié ^5^, Yann Neuzillet ^2^, Xavier Paoletti ^1*^, ThierryLebret ^2^

^1^ Service de Biostatistique et d'Epidémiologie & CESP OncoStat, INSERM, Institut Gustave Roussy, Université Paris Saclay, UVSQ, Villejuif, France.

^2^ Department of Urology, Hôpital Foch, Université Parsi-Saclay, Suresnes, France

^3^ INSERM, UMR 946, Genetic Variation and Human Diseases Unit, Paris, France

^4^ Department of Pathology, Hôpital Foch, Université Paris-Sacaly, Suresnes, France

^5^ Department of Pathology, University Hospital Centre of Martinique, France

**Figure A: Hazard functions for recurrence (A.1), progression to Ta/T1 stage (A.2) or progression to muscle invasive bladder cancer (A.3)**

Fig A represents the hazard function for the first recurrence, which is the rate at which new events occur in the at-risk population; it was maximal before one year after diagnosis and then decreased progressively over time; at three years, the cumulative incidence of recurrence was close to 0.7. Fig B represents the hazard function for the progression to Ta/T1 stage bladder cancer or non-muscle invasive bladder cancer (NMIBC); it decreased continuously over time. Fig C represents the hazard function for the progression to muscle invasive bladder cancer (MIBC). The hazard risk of muscle invasive bladder cancer progression was low in the first year after diagnosis and then increased thereafter.

**
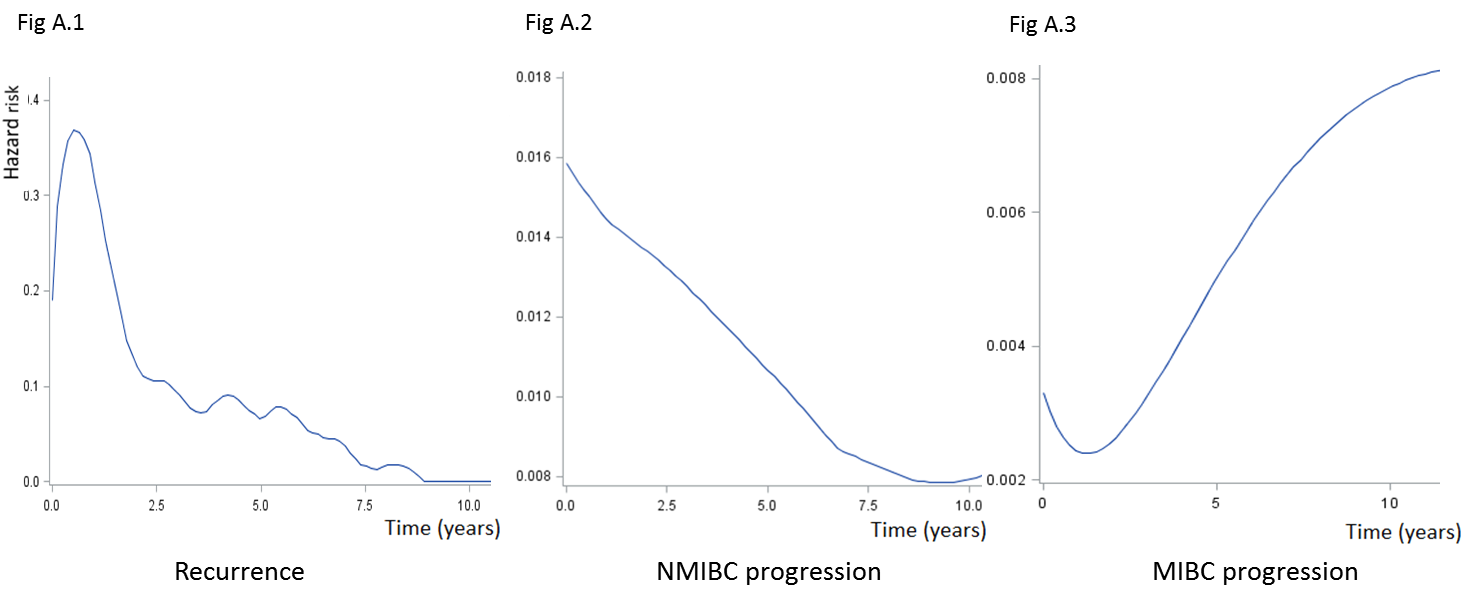
**

**Table A. Sensitivity analysis of the risk of progression after exclusion of the 20 patients who relapsed within the 3 months after the initial TURB.**Table A.1: Multivariate Hazard Ratios (95% Confidence Intervals) of the effect of the number of recurrences on both progression-free time with details on covariates–Variable time-dependant cox model. Table A.2: Multivariate Hazard Ratios (95% Confidence Intervals) of the effect of the number of recurrences on NMIBC progression-free time with details on covariates– Number of recurrence is a time-dependant variable in the cox model. Table A.3: Univariate and multivariate analyzes of the effect of the number of recurrences on progression-free survival.

| **Table A.1** | |  | | **BOTH PROGRESSION FREE TIME** ***** | | |
| --- | --- | --- | --- | --- | --- | --- |
|  |  |  | | HR (CI 95%) | | |
|  |  |  | | Univariate | | Multivariate ƚ |
| **Number of recurrences** | 1 | | 1.50 (0.72-3. 11) | | 0.92 (0.41-2.03) | |
|  | 2-3 | | 1.47 (0.64-3.39) | | 0.90 (0.36-2.23) | |
|  | >=4 | | 0.95 (0.31-2.90) | | 0.51 (0.15-1.69) | |
| **Age** | > 67 years | | 1.37 (0.73-2.34) | | 1.40 (0.78-2.51) | |
| **Sex** | Men | | 1.63 (0.64-4.14) | | 1.39 (0.53-3.65) | |
| **Smoking** | Yes | | 1.94 (0.90-4.15) | | 1.79 (0.81-3.95) | |
| **Size** | > 30 mm | | 1.38 (0.70-2.72) | | 1.40 (0.59-3.31) | |
| **Number of tumors** | > 1 | | 0.94 (0.49-1.77) | | - | |
| **Previous treatment** | Yes | | 4.13 (2.17-7.87) | | 4.84 (2.27-10.31) | |

| **EORTC score** | Intermediate /high | 1.09 (0.61-1.93) | - |
| --- | --- | --- | --- |

* Both type of progression: progression to higher stage or grade

Univariate and multivariate hazard ratios were estimated using variable time-dependant cox model.

ƚ Adjusted on age, sex, smoking, previous treatment, and stratified on the EORTC risk score (low versus intermediate / high) at baseline.

| **Table A.2** | |  | | **PROGRESSION TO HIGH GRADE Ta / T1 FREE TIME** ***** | | |
| --- | --- | --- | --- | --- | --- | --- |
|  |  |  | | HR (CI 95%) | | |
|  |  |  | | Univariate | | Multivariate ƚ |
| **Number of recurrences** | 1 | | 2.04 (0.88-4.75) | | 0.90 (0.33-2.46) | |
|  | 2-3 | | 2.66 (1.07-6.61) | | 1.51 (0.54-4.10) | |
|  | >=4 | | 0.91 (0.18-4.52) | | 0.43 (0.07-2.53) | |
| **Age** | > 67 years | | 1.53 (0.77-3.04) | | 1.76 (0.88-3.57) | |
| **Sex** | Men | | 1.18 (0.45-3.04) | | 0.98 (0.35-2.70) | |
| **Smoking** | Yes | | 1.87 (0.78-4.53) | | 1.84 (0.72-4.74) | |
| **Size** | > 30 mm | | 1.17 (0.51-2.68) | | 1.07 (0.39-2.98) | |
| **Number of tumors** | > 1 | | 1.21 (0.59-2.48) | | - | |
| **Previous treatment** | Yes | | 6.40 (3.20-12.80) | | 7.13 (3.01-16.87) | |
| **EORTC score** | Intermediate /high | | 1.08 (0.55-2.13) | | - | |

* NMIBC progression alone.

Univariate and multivariate hazard ratios were estimated using variable time-dependent cox model.

| **Table A.3** | | **BOTH PROGRESSION FREE TIME** ***** | | **PROGRESSION TO HIGH GRADE Ta / T1 FREE TIME** | |  |
| --- | --- | --- | --- | --- | --- | --- |
|  | | HR (CI 95%) | | HR (CI 95%) | |  |
|  |  | Univariate | Multivariate ƚ | Univariate | Multivariate ƚ |  |
| **Number of recurrences** | 1 | 1.50 (0.78-3.38) | 0.97 (0.43-2.19) | 2.20 (0.94-5.17) | 0.92 (0.33-2.55) |  |
|  | 2-3 | 1.47 (0.60-3.46) | 0.88 (0.34-2.26) | 2.64 (1.02-6.85) | 1.39 (0.47-4.07) |  |
|  | >=4 | 1.08 (0.35-3.32) | 0.55 (0.16-1.86) | 1.04 (0.21-5.22) | 0.44 (0.07-2.69) |  |

* Both type of progression: higher stage or grade.

Univariate and multivariate hazard ratios were estimated using variable time-dependent cox model.

ƚ Adjusted on age, sex, smoking, previous treatment, and stratified on the EORTC risk score (low versus intermediate / high) at baseline.

**Table B: Cumulative incidence of first recurrence over the 4 years in patients free of recurrence at increasing timepoints.**

| **Patients free of recurrence at year** | **over years** | **Cumulative incidence** | **95% Confidence Interval** |
| --- | --- | --- | --- |
| 2 | 2 – 6 | 30% | 23 – 37% |
| 3 | 3 – 7 | 24% | 17 – 32% |
| 4 | 4 – 8 | 20% | 14 – 28% |
| 5 | 5 – 9 | 15% | 11 – 25% |

**Table C: Effect of the number of previous recurrences on the risk of subsequent recurrence –Landmark analysis at 3 year.**

| **Landmark – 3 YEARS (n=368)** | |  | | **RECURRENCE FREE TIME** | | |
| --- | --- | --- | --- | --- | --- | --- |
|  |  |  | | HR (CI 95%) | | |
|  |  |  | | Univariate | | Multivariate ƚ |
| **Number of recurrences** | 1 | | 2.21 (1.39-3.51) | | 2.11 (1.28-3.49) | |
|  | 2-3 | | 4.94 (3.25-7.50) | | 4.72 (2.77-8.05) | |
|  | >=4 | | 3.13 (1.39-7.04) | | 2.63 (0.97-7.15) | |
| **Age** | > 67 years | | 0.93 (0.66-1.31) | | 0.96 (0.62-1.51) | |
| **Sex** | Men | | 1.00 (0.64-1.57) | | 1.13 (0.70-1.85) | |
| **Smoking** | Yes | | 0.92 (0.63-1.34) | | 0.79 (0.53-1.18) | |
| **Size** | > 30 mm | | 1.55 (1.03-2.33) | | 1.00 (0.65-1.54) | |
| **Number of tumors** | > 1 | | 1.76 (1.24-2.50) | | 1.25 (0.86-1.82) | |
| **Previous treatment** | Yes | | 0.40 (0.29-0.57) | | 0.96 (0.62-1.51) | |

Univariate and multivariate hazard ratios were estimated using cox model.

**Table D: Multivariate Hazard Ratios (95% Confidence Intervals) of the effect of recurrences’ characteristics on recurrence-free time with details on covariates–Frailty models.**

|  | |  | | **RECUREENCE FREE TIME** | | |
| --- | --- | --- | --- | --- | --- | --- |
|  |  |  | | HR (CI 95%) | | |
|  |  |  | | Univariate | | Multivariate ƚ |
| **Localization** | Multifocal | | 1.53 (1.26-1.86) | | 1.47 (1.19-1.80) | |
| **Age** | > 67 years | | 0.92 (0.79-1.07) | | 0.93 (0.81-1.08) | |
| **Sex** | Men | | 0.91 (0.75-1.11) | | 0.91 (0.75-1.12) | |
| **Smoking** | Yes | | 0.84 (0.72-0.98) | | 0.84 (0.71-0.98) | |
| **Size** | > 30 mm | | 1.05 (0.88-1.24) | | 1.04 (0.88-1.22) | |
| **Number of tumors** | > 1 | | 1.22 (1.05-1.41) | | 1.13 (0.98-1.31) | |
| **Previous treatment** | Yes | | 1.13 (0.96-1.33) | | 1.05 (0.89-1.25) | |

Univariate and multivariate hazard ratios were estimated using Frailty model.

|  | |  | | **RECUREENCE FREE TIME** | | |
| --- | --- | --- | --- | --- | --- | --- |
|  |  |  | | HR (CI 95%) | | |
|  |  |  | | Univariate | | Multivariate ƚ |
| **Time of occurence** | < 6 months | | 1.38 (1.12-1.70) | | 1.33 (1.08-1.63) | |
| **Age** | > 67 years | | 0.92 (0.79-1.07) | | 0.92 (0.76-1.11) | |
| **Sex** | Men | | 0.91 (0.75-1.11) | | 0.90 (0.70-1.16) | |
| **Smoking** | Yes | | 0.84 (0.72-0.98) | | 0.79 (0.64-0.97) | |
| **Size** | > 30 mm | | 1.05 (0.88-1.24) | | 0.99 (0.80-1.23) | |
| **Number of tumors** | > 1 | | 1.22 (1.05-1.41) | | 1.19 (0.98-1.44) | |
| **Previous treatment** | Yes | | 1.13 (0.96-1.33) | | 1.28 (1.05-1.55) | |

Univariate and multivariate hazard ratios were estimated using Frailty model.

**Text A: List of the characteristics of the 47 patients who progressed to higher grade or stage during the follow-up**

| **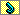Date of diagnosis** | **Age at diagnosis** | **sex** | **Tumour size at diagnosis** | **# of tumours at diag** | **EORTC score** | **# of relapses before 1st Prog** | **Date 1st NMIBC prog** | **pT (1st NMIBC prog)** | **pG (1st** | **Date 1st MIBC prog** | **Ev1** | **Ev2** | **Ev3** | **Ev4** | **Ev5** | **Ev6** | **Ev7** | **Ev8** |
| --- | --- | --- | --- | --- | --- | --- | --- | --- | --- | --- | --- | --- | --- | --- | --- | --- | --- | --- |
|  |  |  |  |  |  |  |  |  | **prog)** |  |  |  |  |  |  |  |  |  |
| 1.7.92 | 62 | M | <30mm | 1 | 0 | 4 |  |  |  | 2.7.11 | relapse | relapse | relapse | relapse | prog MIBC |  |  |  |
| 4.1.90 | 53 | M | <30mm | 1 | 0 | 1 |  |  |  | 29.1.01 | relapse | prog MIBC |  |  |  |  |  |  |
| 15.1.96 | 57 | M | >30mm | 1 | 3 | 5 |  |  |  | 1.3.06 | relapse | relapse | relapse | relapse | relapse | prog MIBC |  |  |
| 20.2.96 | 65 | M | <30mm | 1 | 0 | 0 |  |  |  | 9.2.99 | prog MIBC |  |  |  |  |  |  |  |
| 7.8.96 | 62 | M | >30mm | 1 | 3 | 1 |  |  |  | 3.9.04 | relapse | prog MIBC |  |  |  |  |  |  |
| 25.9.97 | 75 | M | <30mm | 2 to 7 | 3 | 0 |  |  |  | 3.2.98 | prog MIBC |  |  |  |  |  |  |  |
| 7.7.98 | 66 | M | >30mm | 1 | 3 | 1 |  |  |  | 26.8.05 | relapse | relapse | relapse | relapse | prog MIBC |  |  |  |
| 28.9.99 | 77 | M | <30mm | 1 | 0 | 0 |  |  |  | 11.12.08 | prog MIBC |  |  |  |  |  |  |  |
| 10.7.00 | 55 | M | <30mm | 1 | 0 | 0 |  |  |  | 23.12.09 | prog MIBC |  |  |  |  |  |  |  |
| 1.2.02 | 67 | M | <30mm | 1 | 0 | 0 |  |  |  | 24.5.02 | prog MIBC |  |  |  |  |  |  |  |
| 6.11.02 | 57 | M | >30mm | 1 | 3 | 0 |  |  |  | 7.4.05 | prog MIBC |  |  |  |  |  |  |  |
| 21.3.06 | 97 | M | <30mm | 2 to 7 | 3 | 0 |  |  |  | 19.9.06 | prog MIBC |  |  |  |  |  |  |  |
| 9.5.07 | 72 | M | <30mm | 1 | 0 | 0 |  |  |  | 21.5.10 | prog MIBC |  |  |  |  |  |  |  |
| 18.12.95 | 72 | F | >30mm | >7 | 6 | 2 | 27.12.98 | Ta | 3 | 9.4.10 | relapse | relapse | prog NMIBC | relapse | relapse | prog NMIBC | prog MIBC |  |
| 28.10.93 | 69 | M | >30mm | 2 to 7 | 6 | 0 | 4.7.95 | Ta | 2 |  | prog NMIBC |  |  |  |  |  |  |  |
| 15.6.00 | 76 | F | <30mm | 2 to 7 | 3 | 0 | 27.6.01 | Ta | 3 |  | prog NMIBC |  |  |  |  |  |  |  |
| 25.9.01 | 77 | M | <30mm | 1 | 0 | 3 | 5.4.05 | Ta |  |  | relapse | relapse | relapse | prog NMIBC |  |  |  |  |
| 26.9.01 | 61 | M | <30mm | 1 | 0 | 1 | 18.12.09 | Ta |  |  | relapse | prog NMIBC |  |  |  |  |  |  |
| 22.10.01 | 69 | M | <30mm | 2 to 7 | 3 | 0 | 17.5.02 | Ta | 3 |  | prog NMIBC |  |  |  |  |  |  |  |
| 18.2.02 | 75 | F | <30mm | 1 | 0 | 3 | 11.6.08 | Ta |  |  | relapse | relapse | relapse | relapse | prog NMIBC |  |  |  |
| 21.6.01 | 75 | M | <30mm | 1 | 0 | 2 | 6.2.09 | Ta | 3 |  | relapse | relapse | prog NMIBC |  |  |  |  |  |
| 6.11.02 | 70 | M | <30mm | 2 to 7 | 3 | 1 | 14.6.06 | Ta |  |  | relapse | prog NMIBC |  |  |  |  |  |  |
| 19.8.03 | 78 | M | >30mm | 1 | 3 | 1 | 24.5.05 | Ta | 3 | 19.9.08 | relapse | prog NMIBC | prog NMIBC | prog MIBC |  |  |  |  |
| 12.12.01 | 47 | M | <30mm | 1 | 0 | 3 | 13.5.05 | Ta | 2 |  | relapse | relapse | relapse | prog NMIBC |  |  |  |  |
| 4.10.04 | 71 | M | <30mm | 2 to 7 | 3 | 1 | 7.2.07 | Ta | 3 |  | relapse | prog NMIBC |  |  |  |  |  |  |
| 9.4.91 | 63 | M | <30mm | 1 | 0 | 0 | 10.6.97 | T1 | 3 | 1.10.98 | prog NMIBC | relapse | prog MIBC |  |  |  |  |  |
| 17.2.92 | 63 | M | <30mm | 1 | 0 | 0 | 9.9.96 | T1 | 3 |  | prog NMIBC |  |  |  |  |  |  |  |
| 20.5.92 | 72 | M | >30mm | 1 | 3 | 0 | 8.6.94 | T1 | 3 |  | prog NMIBC |  |  |  |  |  |  |  |
| 14.8.92 | 50 | M | <30mm | 1 | 0 | 0 | 29.12.99 | T1 | 2 |  | prog NMIBC |  |  |  |  |  |  |  |
| 18.6.93 | 79 | M | <30mm | 1 | 0 | 0 | 8.8.97 | T1 | 3 |  | prog NMIBC |  |  |  |  |  |  |  |
| 30.9.93 | 60 | M | <30mm | 2 to 7 | 3 | 3 | 17.5.99 | T1 | 2 |  | relapse | relapse | relapse | prog NMIBC |  |  |  |  |
| 6.1.97 | 68 | M | <30mm | 1 | 0 | 2 | 6.5.11 | T1 | 2 |  | relapse | relapse | prog NMIBC |  |  |  |  |  |
| 28.9.97 | 54 | M | <30mm | 1 | 0 | 0 | 31.12.98 | T1 | 3 |  | prog NMIBC |  |  |  |  |  |  |  |
| 6.4.99 | 61 | M | >30mm | 1 | 3 | 0 | 15.5.01 | T1 | 2 |  | prog NMIBC |  |  |  |  |  |  |  |
| 19.7.99 | 75 | M | <30mm | 1 | 0 | 0 | 28.4.00 | T1 | 2 |  | prog NMIBC |  |  |  |  |  |  |  |
| 18.9.00 | 39 | M | >30mm | 1 | 3 | 4 | 27.6.11 | T1 | 3 |  | relapse | relapse | relapse | relapse | prog NMIBC |  |  |  |
| 17.4.01 | 50 | M | <30mm | >7 | 3 | 2 | 10.6.02 | T1 | 3 |  | relapse | relapse | prog NMIBC |  |  |  |  |  |
| 13.3.01 | 66 | M | >30mm | >7 | 6 | 0 | 1.8.01 | T1 | 2 | 26.1.09 | prog NMIBC | prog NMIBC | relapse | prog NMIBC | relapse | relapse | prog NMIBC | prog MIBC |
| 22.3.02 | 78 | F | <30mm | 1 | 0 | 0 | 30.1.03 | T1 | 1 |  | prog NMIBC |  |  |  |  |  |  |  |
| 6.11.00 | 75 | M | <30mm | 1 | 0 | 1 | 6.10.02 | T1 | 2 |  | relapse | prog NMIBC |  |  |  |  |  |  |
| 6.1.03 | 52 | M | <30mm | 2 to 7 | 3 | 1 | 20.6.08 | T1 | 2 |  | relapse | prog NMIBC |  |  |  |  |  |  |
| 26.5.03 | 76 | M | <30mm | 1 | 0 | 2 | 6.8.04 | T1 | 2 |  | relapse | relapse | prog NMIBC |  |  |  |  |  |
| 14.10.98 | 68 | F | <30mm | 1 | 0 | 1 | 5.11.99 | T1 | 3 |  | relapse | prog NMIBC |  |  |  |  |  |  |
| 29.3.99 | 52 | M | <30mm | 1 | 0 | 0 | 31.3.04 | T1 | 3 |  | prog NMIBC |  |  |  |  |  |  |  |
| 1.12.04 | 85 | M | <30mm | 1 | 0 | 0 | 15.3.06 | T1 | 3 |  | prog NMIBC |  |  |  |  |  |  |  |
| 2.9.96 | 59 | M | <30mm | 1 | 0 | 0 | 12.3.02 | T1 | 2 |  | prog NMIBC |  |  |  |  |  |  |  |
| 12.9.08 | 69 | M | <30mm | >7 | 3 | 0 | 3.10.08 | T1 | 1 |  | prog NMIBC |  |  |  |  |  |  |  |
